# Elevated serum uric acid is not an independent risk factor for the occurrence of Type 2 diabetic kidney disease in Chinese populations

**DOI:** 10.1097/MD.0000000000032128

**Published:** 2022-12-16

**Authors:** Lin Zhu, Jiaxing Sun, Xuening Wang, Ruina Tian, Yuexin Zhou, Jiangyi Yu, Xiaofei An

**Affiliations:** a Department of Endocrinology, Affiliated Hospital of Nanjing University of Chinese Medicine, Nanjing, Jiangsu, China.

**Keywords:** albuminuria, diabetic kidney disease, serum uric acid, type 2 diabetes

## Abstract

Previous studies suggested that increased serum uric acid (SUA) level is an independent risk factor for albuminuria in Type 2 diabetes (T2D) patients. However, the association between SUA and onset of Type 2 DKD (T2DKD) remained to be clarified. This was a cross-sectional clinical study in which 1210 Chinese T2D patients were enrolled. According to the urine albumin-to-creatinine ratio (UACR), the cohort was divided into normal-albuminuria (UACR < 30 mg/g), micro-albuminuria (UACR 30–300 mg/g) and macro-albuminuria (UACR > 300 mg/g). The micro- and macro-albuminuria groups were combined into albuminuria category. Results showed that T2D patients with macro-albuminuria have significantly higher SUA than the other 2 groups (*P* < .001). In the binary logistic regression model, the subjects with SUA higher than 420 μmol/L were associated with a 2-fold increase in the odds of albuminuria (odds ratio = 2.024, 95% confidence interval: 1.232–3.325, *P* = .005), as compared with those with SUA lower than 300 μmol/L. Moreover, the multinomial regression analysis revealed that the subjects with SUA higher than 420 μmol/L had about 3-fold increase in the odds of macro-albuminuria (odds ratio = 3.758, 95% confidence interval: 2.051–6.885, *P* < .001), as compared with those with SUA lower than 300 μmol/L. However, SUA was not significantly associated with the presence of micro-albuminuria. Although the SUAwas not independently risk factor for micro-albuminuria, it was closely correlated with the development of macro-albuminuria in Chinese T2DKD patients. Elevated SUA may be useful for predicting the occurrence of macro-albuminuria but not onset of micro-albuminuria at the early stage of T2DKD.

## 1. Introduction

Diabetic kidney disease (DKD) is regarded as the leading cause of chronic kidney disease (CKD) and end-stage renal disease worldwide, occurring in approximately 40% of individuals with Type 2 diabetes (T2D) and 30% of those with Type 1 diabetes (T1D).^[1,2]^ DKD is characterized with persistent albuminuria and progressive decline of renal function.^[2]^ Reciprocally, persistent albuminuria is an independent risk factor for DKD progression.^[3]^ Moreover, evidence from multiple observational studies and clinical trials in diabetes has suggested that albuminuria acts as a strong predictor of renal and cardiovascular events.^[4,5]^

Elevated serum uric acid (SUA), a final product of purine nucleotide metabolism, may reflect endothelial dysfunction, activation of renin-angiotensin-aldosterone system (RAAS), increase of oxidative stress and inflammation.^[6–8]^ Elevated SUA level might contribute to the pathogenesis of diabetes and its complications.^[9]^ In patients with T1D, the association between elevated SUA level and the development and progression of albuminuria has been reported by several studies.^[10–12]^ However, a cross-sectional study from Steno Diabetes Center Copenhagen failed to show the significant association in Caucasian T1D patients after adjustment included sex, age, diabetes duration, body mass index, high-density, lipoprotein cholesterol, smoking, hemoglobin A1c (HbA1_C_), 24-hours pulse pressure, estimated glomerular filtration rate (eGFR) and treatment with RAAS blockers.^[13]^ The association between elevated SUA and albuminuria in patients with T2D also remained controversial. Some observational studies have shown that elevated level of SUA was associated with increased risk of the development of albuminuria,^[14,15]^ whereas a large prospective cohort study based on a Japanese diabetes registry reported a significant relationship between baseline SUA level and progression from micro- to macro-albuminuria, not the onset of micro-albuminuria in patients with T2D.^[16]^ We conducted a cross-sectional study to examine the association of SUA levels with micro-albuminuria and macro-albuminuria in Chinese patients with T2D.

## 2. Materials and Methods

### 2.1. Participants

The study was conducted in the Department of Endocrinology, Affiliated Hospital of the Nanjing University of Chinese Medicine. This clinical study was approved by the local ethics committee of the Affiliated Hospital of Nanjing University of Chinese Medicine (2019NL-I09-02), and registered in the Chinese Clinical Trial Registry (ChiCTR2000028949). A total of 1210 patients with Type 2 diabetes were consecutively recruited from our diabetes patient ward for medical examination and complications screening. Patients with acute diabetic complications, urinary tract infection, cancer or any other serious chronic debilitating disease were excluded.

### 2.2. Data collection and procedures

Demographic parameters were collected and glucose and lipids metabolic index were measured. Data including diabetes duration, body mass index, systolic and diastolic blood pressure, medications, hyperuricemia and hypertension were also collected systematically. All biochemical indexes were measured in our clinical laboratory with ISO15189 certification. SUA and serum creatinine was measured by Beckman automatic biochemical analyzer in the fasting fresh blood samples obtained from the subjects between 8:00 and 9:00 am. Urinary albumin was measured by an immunonephelometric method on an early morning spot sample and expressed as the albumin-to-creatinine ratio.

### 2.3. Definitions

Type 2 diabetic kidney disease (T2DKD) was diagnosed according to the KDOQI guidelines.^[17]^ The eGFR was calculated by the Chronic Kidney Disease Epidemiology Collaboration equation.^[18]^ Hyperuricemia was defined as SUA at > 420 μmol/L (7 mg/dL). Dyslipidemia was defined as TC ≥ 5.18 mmol/L, TG ≥ 1.7 mmol/L, LDL-C ≥ 3.37 mmol/L or HDL-C < 1.04 mmol/L.^[19]^ Target blood pressure was 130/80 mm Hg.^[20]^ Target of HbA1c was from < 6.5% to < 8.0% based on eGFR, macrovascular complications, comorbidities, life expectancy, hypoglycemia awareness, resources for hypoglycemia management and propensity of treatment to cause hypoglycemia.^[17]^ Blood biochemistry was conducted using an automatic biochemical analyzer (Beckman Coulter, CA, USA). Urine albumin and creatinine (UACR) were measured using a Beckman Coulter AU680 (Brea, USA). Normal- albuminuria was defined as UACR ＜30 mg/g. Micro-albuminuria was defined as UACR 30–300 mg/g. Macro-albuminuria was defined as UACR ＞300 mg/g.

### 2.4. Statistical analysis

Continuous variables following normal distribution are presented as mean ± standard deviation, and analyzed using 1-way ANOVA, followed by Tukey post hoc analysis for pairwise comparisons. Continuous variables not following normal distribution are presented as median and interquartile range, and analyzed using Kruskal–Wallis test. Categorical variables are presented as percentage and analyzed using chi-square tests. Multivariate regression analysis was conducted to examine the risk factors for micro- and macro-albuminuria. Factors entered as candidate independent variables included demographics (age and sex) and all other factors associated with the outcome in the univariate analysis, except for variables with multicollinearity correlations.

Participants were further stratified according to SUA levels: reference value, < 300 μmol/L (5 mg/dL); low, 300–360 μmol/L (6 mg/dL); moderate, 360–420 μmol/L (7 mg/dL); and high > 420 µmol/L. A sensitivity analysis was used to verify the association of SUA categories with micro- and macro-albuminuria. The results of regression analysis are reported as odds ratio (OR) and 95% confidence interval (95% CI).

All statistical analyses were conducted using SPSS 26.0 (IBM, Armonk, NY). Statistical significance was as *P* < .05 (2-tailed).

## 3. Results

### 3.1. Characteristics of the study participants

The analysis included a total of 1210 patients with T2D (mean age 57.2 ± 11.9 years, median disease duration 10 years): 452 patients with no albuminuria, 450 with micro-albuminuria and 308 with macro-albuminuria (Table 1). The mean SUA was 302 ± 88 μmol/L in the normal-albuminuria, 310 ± 98 μmol/L in the micro-albuminuria and 382 ± 121 μmol/L in the macro-albuminuria groups, respectively (*P* < .001). Pairwise comparison revealed that significantly higher SUA in patients with macro-albuminuria than with no or micro-albuminuria (*P* < .001 for both) but no significant difference between the normal-albuminuria and micro-albuminuria groups. The prevalence of hyperuricemia was significantly higher in the macro-albuminuria group, compared with the normal-albuminuria (33.8% vs 8.6%, *P* < .001) and micro-albuminuria (33.8% vs 13.1%, *P* < .001) groups. In the micro-albuminuria group, hyperuricemia was significantly higher than that in the normal-albuminuria group (13.1% vs 8.6%, *P* < .001).

**Table 1:** Characteristics of patients with Type 2 diabetes grouped by Urine albumin and creatinine values.

Variables	All patients (n = 1210)			T2DM		*P* 1	*P* 2
		No albuminuria (n = 452)	Albuminuria (n = 758)	Micro-albuminuria (n = 450)	Macro-albuminuria (n = 308)		
Clinical characteristics							
Age (years)	57.2 ± 11.9	55.4 ± 10.8	58.3 ± 12.4	56.6 ± 12.7	60.7 ± 11.6^bc^	< 0.001	0.02
Male (n, %)	744 (61.5)	270 (59.7)	474 (62.5)	272 (60.4)	202 (65.6)	0.24	0.22
Diabetes duration (years)	10 (4–16)	7 (2–12)	10 (5–18)	9 (3–15)^a^	15 (10–20)^bc^	< 0.001	< 0.001
Hypertension (n, %)	898 (74.2)	274 (60.6)	624 (82.3)	348 (77.3)^a^	276 (89.6)^bc^	< 0.001	< 0.001
Dyslipidemia (n, %)	832 (69.3)	308 (68.3)	524 (69.9)	317 (71.1)	207 (68.1)	0.49	0.58
Hyperuricemia (n, %)	202 (16.7)	39 (8.6)	163 (21.5)	59 (13.1)^a^	104 (33.8)^bc^	< 0.001	< 0.001
BMI (kg/m^2^)	25.5 ± 3.7	25.5 ± 3.5	25.4 ± 3.9	25.4 ± 4.0	25.5 ± 3.7	0.76	0.91
Biochemical variables							
eGFR (mL/minute/1.73 m^2^)	89.3 ± 26.1	98.8 ± 15.4	83.7 ± 9.4	95.4 ± 22.9^a^	66.6 ± 29.5^bc^	< 0.001	< 0.001
SUA (μmol/L)	325 ± 106	302 ± 88	339 ± 114	310 ± 98	382 ± 121^bc^	< 0.001	< 0.001
FPG (mmol/L)	8.36 ± 3.36	7.58 ± 2.79	8.82 ± 3.58	9.03 ± 3.56^a^	8.51 ± 3.58^c^	< 0.001	< 0.001
HbA1c (%)	8.9 ± 2.2	8.5 ± 2.1	9.1 ± 2.2	9.3 ± 2.2^a^	8.8 ± 2.2^b^	< 0.001	< 0.001
Anti-diabetic drugs							
Metformin (n, %)	357 (29.5)	149 (33.0)	208 (27.4)	148 (32.9)	60 (19.5)^bc^	0.01	< 0.001
Sulfonylureas (n, %)	310 (25.6)	143 (31.7)	167 (22.0)	109 (24.2)^a^	58 (18.8)	< 0.001	< 0.001
Insulin (n, %)	619 (51.1)	151 (33.4)	468 (61.7)	252 (56.0)^a^	216 (70.1)^bc^	< 0.001	< 0.001
Anti-hypertension drugs							
ACE-I/ARB (n, %)	383 (31.7)	113 (25.0)	270 (35.6)	136 (30.2)	134 (43.5)^bc^	< 0.001	< 0.001
CCB (n, %)	410 (33.9)	115 (25.5)	295 (38.9)	138 (30.7)	157 (51.0)^bc^	< 0.001	< 0.001
Diuretics (n, %)	97 (8.0)	31 (6.9)	66 (8.7)	26 (5.8)	40 (13.0)^bc^	0.16	0.001
Target attainment rate of glucose (n, %)	247 (21.9)	107 (25.2)	140 (20.0)	63 (15.0)^a^	77 (27.3)^b^	0.02	< 0.001
Target attainment rate of blood pressure (n, %)	588 (48.6)	271 (60.0)	317 (41.8)	213 (47.3)^a^	104 (33.8)^bc^	< 0.001	< 0.001

ACEI = angiotensin converting enzyme inhibitor, ARB = angiotensin receptor antagonist, BMI = body mass index, CCB = calcium channel blocker, eGFR = estimated glomerular filtration rate, FPG = fasting plasma glucose, HbA1c = glycosylated hemoglobin A1c, SUA = serum uric acid, T2DM = type 2 diabetic mellitusP1 Comparison between the normal-albuminuria and the albuminuria group.

P2 Comparison among the norm-albuminuria, the micro-albuminuria, and the macro-albuminuria groups by 1-way ANOVA.

a Comparing the normal-albuminuria with the micro-albuminuria group.

b Comparing the micro-albuminuria with the macro-albuminuria group.

c Comparing the normal-albuminuria with the macro-albuminuria group.

### 3.2. Comparison between the normal and albuminuria groups

We first conducted an analysis combining micro- and macro-albuminuria into an albuminuria group (Table 1). Compared with the normal-albuminuria group, the subjects with albuminuria had significantly older age, longer diabetes duration, higher fasting blood glucose, higher HbA1c, and higher rate of hypertension. The albuminuria group also had lower eGFR and lower target attainment rate of glucose and blood pressure. SUA was 339 ± 114 vs. 302 ± 88 μmol/L in subjects with vs. without albuminuria (*P* < .001). Hyperuricemia was higher in subjects with (21.5%) vs. without albuminuria (8.6%, *P* < .001).

### 3.3. Pairwise comparisons among the no, micro- and macro-albuminuria groups

The pairwise comparisons among the normal-, micro- and macro-albuminuria groups (Table 1) were then conducted. Compared with the normal-albuminuria group, the subjects with micro-albuminuria or macro-albuminuria had significantly longer diabetes duration, lower eGFR, higher fasting blood glucose and higher rate of hypertension. Moreover, the subjects with micro-albuminuria had significantly highest HbA1c and lowest target attainment rate of glucose among the 3 groups. In addition, the subjects with macro-albuminuria had significantly oldest age, longest diabetes duration, lowest eGFR, highest prevalence of hypertension, lowest target attainment rate of blood pressure, highest rate of insulin and anti-hypertensive drugs treatment.

### 3.4. The association of SUA with albuminuria in T2D

Table 2 highlighted the unadjusted and adjusted analysis using a binary logistic regression model. Variables with statistical significance in the unadjusted analysis included age, eGFR, SUA, diabetes duration, glucose, hypertension, percentage of anti-diabetic or anti-hypertension drugs usage and target attainment rate of blood pressure and glucose. In the adjusted analysis, SUA level remained to be significantly correlated with albuminuria in our cohort (OR = 1.002, 95% CI: 1.000–1.004, *P* = .02).

**Table 2:** Binary logistic regression of factors associated with albuminuria in patients with Type 2 diabetes.

	Unadjusted		Adjusted	
	OR (95% CI)	*P*	OR (95% CI)	*P*
Clinical characteristics				
Age (per 1 year)	1.020 (1.010–1.030)	< 0.001	1.021 (1.004–1.039)	0.02
Male gender (versus female)	1.125 (0.886–1.428)	0.33	1.015 (0.750–1.372)	0.93
Log duration of DM (per 1 year)	1.065 (1.047–1.084)	< 0.001	1.027 (1.004–1.050)	0.02
Hypertension	3.025 (2.320–3.945)	< 0.001	1.726 (1.110–2.683)	0.02
Dyslipidemia	1.076 (0.836–1.385)	0.58	–	–
BMI (per 1 kg/m^2^)	0.994 (0.964–1.026)	0.73	–	–
Biochemical variables				
eGFR (per1 mL/minute/1.73 m^2^)	0.974 (0.968–0.979)	< 0.001	0.971 (0.962–0.981)	< 0.001
SUA (per 1 µmol/L)	1.004 (1.002–1.005)	< 0.001	1.002 (1.000–1.004)	0.02
FPG (per 1 mmol/L)	1.130 (1.087–1.175)	< 0.001	1.153 (1.089–1.220)	< 0.001
HbA1c (per1%)	1.122 (1.059–1.188)	< 0.001	1.130 (1.029–1.241)	0.01
Treatment				
Anti-diabetic drugs				
Metformin	0.767 (0.595–0.987)	0.04	0.826 (0.605–1.128)	0.23
Sulfonylureas	0.609 (0.468–0.791)	< 0.001	0.807 (0.588–1.107)	0.18
Insulin	3.217 (2.520–4.107)	< 0.001	2.363 (1.751–3.188)	< 0.001
Anti-hypertension drugs				
ACEI/ARB	1.660 (1.280–2.152)	< 0.001	0.947 (0.650–1.380)	0.78
CCB	1.862 (1.439–2.408)	< 0.001	1.147 (0.791–1.663)	0.47
Diuretics	1.292 (0.829–2.014)	0.26	–	–
Target attainment rate of glucose	0.742 (0.557–0.988)	0.04	1.009 (0.648–1.569)	0.97
Target attainment rate of blood pressure	0.480 (0.379–0.609)	< 0.001	0.710 (0.496–1.016)	0.06

ACEI = angiotensin converting enzyme inhibitor, ARB = angiotensin receptor antagonist, BMI = body mass index, CCB = calcium channel blocker, DM = diabetes duration, eGFR = estimated glomerular filtration rate, FPG = fasting plasma glucose, HbA1c = glycosylated hemoglobin A1c, SUA = serum uric acid.

### 3.5. The association of SUA with micro-albuminuria or macro-albuminuria

The multinomial logistic regression analysis (Tables 3 and 4) shows association between SUA and the presence of micro- and macro-albuminuria in T2D. Table 3 highlights that there was no statistically significance of SUA in both the unadjusted (OR = 1.001, 95% CI: 1.000–1.002, *P* = .18) and adjusted analyses (OR = 1.001, 95% CI: 0.999–1.003, *P* = .24) when comparing the no and micro-albuminuria groups.

**Table 3:** Multinomial logistic regression of factors associated with micro-albuminuria in patients with Type 2 diabetes.

	Micro-albuminuria^a^			
	Unadjusted		Adjusted	
	OR (95% CI)	*P*	OR (95% CI)	*P*
Clinical characteristics				
Age (per 1 year)	1.008 (0.997–1.019)	0.14	1.006 (0.988–1.025)	0.50
Male gender (versus female)	1.030 (0.789–1.345)	0.83	1.035 (0.756–1.417)	0.83
Log duration of DM (per 1 year)	1.030 (1.010–1.050)	0.004	1.004 (0.980–1.029)	0.75
Hypertension	2.216 (1.658–2.963)	< 0.001	1.802 (1.177–2.759)	0.007
Dyslipidemia	1.141 (0.858–1.517)	0.37	–	–
BMI (per 1 kg/m^2^)	0.992 (0.958–1.028)	0.66	–	–
Biochemical variables				
eGFR (per1 mL/minute/1.73 m2)	0.991 (0.985–0.998)	0.01	0.987 (0.976–0.998)	0.02
SUA (per1 μmol/L)	1.001 (1.000–1.002)	0.18	1.001 (0.999–1.003)	0.24
FPG (per 1 mmol/L)	1.149 (1.101–1.199)	< 0.001	1.138 (1.073–1.206)	< 0.001
HbA1c (per1%)	1.166 (1.094–1.243)	< 0.001	1.103 (1.000–1.216)	0.05
Treatment				
Anti-diabetic drugs				
Metformin	0.993 (0.752–1.311)	0.96	–	–
Sulfonylureas	0.688 (0.514–0.923)	0.01	0.809 (0.582–1.125)	0.21
Insulin	2.713 (2.123–3.466)	< 0.001	2.368 (1.781–3.149)	< 0.001
Anti-hypertension drugs				
ACE-I/ARB	1.299 (0.969–1.742)	0.08	–	–
CCB	1.287 (0.964–1.719)	0.09	–	–
Diuretics	0.831 (0.485–1.423)	0.50	–	–
Target attainment rate of glucose	0.526 (0.372–0.743)	< 0.001	0.992 (0.619–1.591)	0.97
Target attainment rate of blood pressure	0.600 (0.461–0.782)	< 0.001	0.821 (0.564–1.195)	0.30

ACEI = angiotensin converting enzyme inhibitor, ARB = angiotensin receptor antagonist, BMI = body mass index, CCB = calcium channel blocker, DM = diabetes duration, eGFR = estimated glomerular filtration rate, FPG = fasting plasma glucose, HbA1c = glycosylated hemoglobin A1c, SUA = serum uric acid.

a Reference category: normal-albuminuria.

**Table 4:** Multinomial logistic regression of factors associated with macro-albuminuria in patients with Type 2 diabetes.

	Macro-albuminuria^a^				Macro-albuminuria^b^			
	Unadjusted		Adjusted		Unadjusted		Adjusted	
	OR (95% CI)	*P*	OR (95% CI)	*P*	OR (95% CI)	*P*	OR (95% CI)	*P*
Clinical characteristics								
Age (per 1 year)	1.040 (1.027–1.054)	<0.001	1.046 (1.023–1.069)	<0.001	1.032 (1.018–1.045)	<0.001	1.041 (1.020–1.062)	<0.001
Male gender (vs female)	1.285 (0.951–1736)	0.10	1.059 (0.701–1.599)	0.79	1.274 (0.922–1.686)	0.151	1.066 (0.717–1.586)	0.75
Log duration of DM (per 1 year)	1.121 (1.097–1.146)	<0.001	1.071 (1.041–1.102)	<0.001	1.089 (1.067–1.112)	<0.001	1.070 (1.041–1.100)	< 0.001
Hypertension	5.603 (3.712–8.458)	<0.001	1.400 (0.728–2.694)	0.31	2.528 (1.649–3.876)	<0.001	0.609 (0.317–1.170)	0.14
Dyslipidemia	0.991 (0.725–1.354)	0.95	–	–	0.868 (0.633–1.192)	0.382	–	–
BMI (per 1 kg/m^2^)	0.998 (0.960–1.037)	0.90	–	–	1.005 (0.967–1.045)	0.785	–	–
Biochemical variables								
eGFR (per1 mL/minute/1.73 m^2^)	0.948 (0.941–0.955)	<0.001	0.952 (0.941–0.963)	<0.001	0.956 (0.949–0.963)	<0.001	0.963 (0.953–0.972)	< 0.001
SUA (per 1 µmol/L)	1.008 (1.006–1.009)	<0.001	1.004 (1.002–1.006)	<0.001	1.007 (1.005–1.008)	<0.001	1.003 (1.001–1.005)	0.003
FPG (per 1 mmol/L)	1.102 (1.051–1.155)	<0.001	1.204 (1.133–1.279)	<0.001	0.959 (0.919–1.000)	0.048	1.019 (0.959–1.082)	0.54
HbA1c (per1%)	1.059 (0.982–1.135)	0.14	–	–	0.905 (0.844–0.970)	0.005	1.039 (0.924–1.169)	0.52
Treatment								
Anti-diabetic drugs								
Metformin	0.500 (0.353–0.708)	<0.001	0.541 (0.351–0.832)	0.005	0.494 (0.350–0.696)	<0.001	0.713 (0.470–1.081)	0.11
Sulfonylureas	0.688 (0.514–0.923)	<0.001	0.775 (0.498–1.208)	0.26	0.726 (0.507–1.038)	0.079	–	–
Insulin	4.089 (3.120–5.359)	<0.001	2.518 (1.765–3.594)	<0.001	1.507 (1.185–1.918)	0.001	0.961 (0.698–1.324)	0.81
Anti-hypertension drugs								
ACEI/ARB	2.310 (1.695–3.150)	<0.001	1.049 (0.647–1.701)	0.85	1.778 (1.315–2.405)	<0.001	1.131 (0.723–1.770)	0.59
CCB	3.086 (2.277–4.181)	<0.001	1.699 (1.076–2.684)	0.02	2.398 (1.785–3.221)	<0.001	1.921 (1.256–2.937)	0.003
Diuretics	2.022 (1.235–3.312)	0.005	0.808 (0.417–1.565)	0.53	2.434 (1.452–4.081)	0.001	1.699 (0.885–3.261)	0.11
Target attainment rate of glucose	1.116 (0.793–1.571)	0.53	–	–	2.122 (1.459–3.087)	<0.001	0.854 (0.472–1.543)	0.60
Target attainment rate of blood pressure	0.340 (0.252–0.461)	<0.001	0.463 (0.290–0.739)	0.001	0.567 (0.420–0.766)	<0.001	0.503 (0.323–0.785)	0.002

ACEI = angiotensin converting enzyme inhibitor, ARB = angiotensin receptor antagonist, BMI = body mass index, CCB = calcium channel blocker, DM = diabetes duration, eGFR = estimated glomerular filtration rate, FPG = fasting plasma glucose, HbA1c = glycosylated hemoglobin A1c, SUA = serum uric acid.

a Reference category: normal-albuminuria.

b Reference category: micro-albuminuria.

Taking normal-albuminuria or micro-albuminuria as the reference category respectively, the risk factors for macro-albuminuria were demonstrated in Table 4. In the unadjusted analysis, variables with statistical significance were consistent with the unadjusted analysis in the binary regression analysis, especially when comparing micro- albuminuria with macro-albuminuria. The association between SUA levels and macro-albuminuria persisted after multivariable adjustment (OR = 1.004, 95% CI: 1.002–1.006, *P* < .001, using normal-albuminuria as the reference category, OR = 1.003, 95% CI: 1.001–1.005, *P* = .003, using micro-albuminuria as the reference category).

### 3.6. Sensitivity analysis

Sensitivity analysis confirmed that higher SUA categories were associated with albuminuria and macro-albuminuria in T2D as accurately as SUA level did (Tables 5 and 6). Similarly, a higher SUA category was not associated with micro-albuminuria in T2D (Table 7).

**Table 5:** Sensitivity analysis to identify the association of SUA level with albuminuria in patients with Type 2 diabetes based on binary logistic regression.

	Unadjusted		Adjusted	
SUA (µmol/L)	OR (95% CI)	*P*	OR (95% CI)	*P*
<300	1 (reference)		1 (reference)	
300-360	1.337 (0.991–1.804)	0.06	1.308 (0.906–1.887)	0.15
360-420	1.614 (1.155–2.256)	0.005	1.258 (0.823–1.924)	0.29
>420	3.447 (2.336–5.086)	< 0.001	2.024 (1.232–3.325)	0.005

SUA = serum uric acid.

**Table 6:** Sensitivity analysis to identify the association of serum uric acid level with micro-albuminuria in patients with Type 2 diabetes based on multinomial logistic regression.

SUA (µmol/L)	Micro-albuminuria^g^			
	Unadjusted		Adjusted	
	OR (95% CI)	*P*	OR (95% CI)	*P*
<300	1 (reference)		1 (reference)	
300–360	1.149 (0.828–1.594)	0.41	1.252 (0.854–1.833)	0.25
360–420	1.116 (0.764–1.631)	0.57	1.156 (0.736–1.814)	0.53
>420	1.689 (1.083–2.634)	0.02	1.598 (0.943–2.709)	0.08

SUA, serum uric acid.

g Reference category: normal-albuminuria.

**Table 7:** Sensitivity analysis to identify the association of serum uric acid level with macro-albuminuria in patients with Type 2 diabetes based on multinomial logistic regression.

SUA (µmol/L)	Macro-albuminuria^a^				Macro-albuminuria^b^			
	Unadjusted		Adjusted		Unadjusted		Adjusted	
	OR (95% CI)	*P*	OR (95% CI)	*P*	OR (95% CI)	*P*	OR (95% CI)	*P*
<300	1 (reference)				1 (reference)			
300–360	8.421 (5.373–13.199)	< 0.001	1.441 (0.866–2.399)	0.16	4.987 (3.300–7.536)	< 0.001	1.198 (0.728–1.973)	0.48
360–420	3.023 (1.980–4.613)	< 0.001	1.655 (0.954–2.869)	0.07	2.708 (1.770–4.142)	< 0.001	1.428 (0.829–2.458)	0.20
>420	1.870 (1.243–2.814)	0.003	3.758 (2.051–6.885)	< 0.001	1.628 (1.081–2.452)	0.02	2.039 (1.169–3.556)	0.01

SUA, serum uric acid.

a Reference category: normal-albuminuria.

b Reference category: micro-albuminuria.

The T2D patients with SUA higher than 420 μmol/L were associated with a 2-fold increase in the odds of albuminuria (OR = 2.024, 95% CI: 1.232–3.325, *P* = .005), as compared with the subjects with SUA lower than 300 μmol/L. The subjects with SUA more than 420 μmol/L (7 mg/dL) were associated with 2 or 3-fold increase in the odds of macro-albuminuria, as compared with those SUA lower than 300 μmol/L (5 mg/dL) (OR = 3.665, 95% CI: 1.979–6.786, *P* < .001, using normal-albuminuria as the reference category, OR = 2.034 95% CI: 1.166–3.547, *P* = .01, using micro-albuminuria as the reference category).

## 4. Discussion

To the best of our knowledge, our study firstly evaluated the relationship between SUA level and the presence of micro-albuminuria and macro-albuminuria in a large Chinese cohort of patients with T2D. We found that increased SUA was an independent predicative risk factor for albuminuria in T2D patients. The further analysis according to different level of albuminuria suggested that SUA was not associated with the presence of micro-albuminuria but significantly correlated with macro-albuminuria in the T2D cohort. Our findings suggested that the elevated SUA level is not an independently risk factor for the early onset of DKD in T2D patients. However, the increased SUA level might be the significant predicator for development of micro-albuminuria to macro-albuminuria in advanced T2DKD.

In recent years, increasing attention has been paid to the association between SUA and T2DKD. However, the role of SUA in the onset of DKD remained still controversial. Several cohort studies have demonstrated that increased SUA level is regarded as an independent risk factor for the development of albuminuria.^[14,15]^ For example, a cross-sectional study in Taiwan displayed that there was close relationship between increased SUA level and the risk of albuminuria in a cohort containing 385 T2D subjects.^[14]^ Each 1mg/dL (60 μmol/L) increase in SUA level was accompanied by 22.7% increase in the odds of albuminuria even after the adjustment of eGFR, sex, age, diabetes duration, fasting glucose and HbA1c at baseline. In addition, a prospective study from Japan showed that SUA was an independent risk factor for the onset of albuminuria despite the association was weak.^[15]^ For every 1 μmol/L higher SUA at baseline, T2D patients had 1-fold increased risk of developing albuminuria during a median follow up period of 6.9 years. However, in the above several studies, this positive association of SUA and albuminuria in T2D patients has failed to be confirmed.^[16,21]^ For example, another prospective cohort study based on a Japanese diabetes registry involving 2518 patients failed to observe the correlation between SUA and the onset of albuminuria after 2 years of follow-up.^[16]^ The associations between SUA level and the risk of micro-albuminuria with special focus on Chinese Type 2 DKD patients have not been investigated. Our present research offered evidence that SUA level was not an independent risk factor for the early onset of DKD in T2D patients.

Interestingly, it was found in our study that elevated SUA was closely correlated with the development of macro-albuminuria in patients with T2DKD. This was similar to 1 cohort study in Japan in which the baseline SUA levels were demonstrated to be a significant and independent predictor for progression from micro-albuminuria to macro-albuminuria in T2D patients.^[16]^ However, our results were inconsistent with the prospective data from Kailuan cohort.^[22]^ The community-based study in China involving 1327 diabetic patients failed to reveal an association between high level of SUA and development of macro-albuminuria with 10 years follow-up period, though an increase trend of cumulative incidence of macro-albuminuria. The possible reason for the conflicting results might be due to a lower statistical power caused by a lower proportion of patients with hyperuricemia (5.3%) in the Kailuan study.^[22]^ Besides, Type 1 and Type 2 diabetes were not distinguished in the study. In addition, our finding offered the first evidence that the subjects with SUA higher than 420 μmol/L (7 mg/dL) was associated with 3-fold increase in the odds of macro-albuminuria in T2D patients, as compared with those SUA lower than 300 μmol/L (5 mg/dL). Admittedly, further studies are needed to identify an optimal range of SUA among patients with T2DKD in Chinese population.

Although the causal relationship between SUA and DKD has not been clarified, evidence from experimental studies explored the molecular mechanism underlying the close relationship of SUA levels with progression of renal injury. Elevated SUA levels could cause activation of RAAS, endothelial dysfunction, oxidative stress and inflammation at the cellular and tissue level which was associated with glomerulosclerosis and tubulointerstitial fibrosis in kidney.^[6,8]^ Normalizing SUA levels could attenuate the progression of albuminuria and renal structural damages in T2D mice model.^[23]^ Furthermore, the association between CKD progression and SUA has been investigated in human biopsies. It was shown that CKD patients with high levels of SUA displayed more serious segmental glomerulosclerosis and tubular atrophy/interstitial fibrosis.^[24]^ In addition, the renal beneficial effects (improvement in eGFR and decline in albuminuria) have been observed in T2D by lowering SUA therapy.^[25–27]^ However, these studies are small sample size, open labeled and limited generalizability due to the enrolled populations and research conditions. It remained unclear about the important role of SUA in the pathogenesis of T2DKD till now. Further studies are needed to clarify the benefit of lowering SUA therapy to prevent and slow down the kidney damage of T2D.

In consistent with the previous studies,^[28,29]^ our study indicated that age, high glucose levels, duration of diabetes and hypertension of patients were the independent risk factors for the development of T2DKD. Most of these risk factors could be controllable by anti-diabetic or anti-hypertensive treatment and lifestyle changes. In addition, our study demonstrated intensive blood pressure control could reduce the risk of proteinuria, especially in those patients with preexisting macro-albuminuria, which was in accordance with Diabetes Control and Complications Trial/Epidemiology of Diabetes Interventions and Complications Study.^[30]^ Both the United Kingdom Prospective Diabetes study and the DCCT studies suggest that intensive blood glucose control (HbA1c ≤ 7%) could significantly reduce the risk of the onset and progression of albuminuria in patients with diabetes.^[30,31]^ However, there was not a significant association between target attainment rate of glucose and the postponed development of albuminuria in our study. The previous meta-analysis showed intensive glucose control increased the number of severe hypoglycemic events but without lower the micro-vascular and macro-vascular events in patients with T2D.^[32]^ Due to the increased risk of hypoglycemia, the intensive glucose control failed to bring cardiovascular benefit, enhanced survival benefit and improved life expectancy to patients in the advanced stage of T2DKD (eGFR ≤ 45 mL/minute).^[17]^ Therefore, T2D patients in our study were stratified with individual HbA1c targets according to eGFR, macrovascular complications, comorbidities, life expectancy, hypoglycemia awareness, resources for hypoglycemia management and propensity of treatment to cause hypoglycemia.^[17]^ However, our study showed that glucose control with individual HbA1c targets was not likely to reduce the risk for the development of albuminuria in our cohort. Future clinical investigation is needed to search for the better strategy for glucose control and optimum HbA1c value for T2DKD patients.

There are several limitations in our present study. First of all, the cross-sectional study could not offer solid evidence for exploring the causal relationship between SUA level and appearance of albuminuria in T2D patients. Secondly, generalization of our results is limited because our cohort was not from multicenter but from homogeneous population at a single center. Thirdly, the usage information of some novel anti-diabetic drugs such as sodium-dependent glucose transporters 2 inhibitor and glucagon-like peptide-1 receptor agonist(s) were not recorded in our data. It was due to the usage rate of those medications was very low in the enrolled subjects.

In conclusions, our study demonstrated that increased SUA level was not an independently risk factor for the onset of micro-albuminuria in the early stage of T2DKD in Chinese patients. However, it was closely correlated with macro-albuminuria and the elevated SUA level could be taken as a significant predictor for the occurrence of macro-albuminuria in the advanced T2DKD.

## Acknowledgements

This work was supported by the National Natural Science Foundation of China (82074359), The National Integrative Medicine Key Training Project of China（2019-44）and The Open Projects of the Discipline of Chinese Medicine of the Nanjing University of Chinese Medicine Supported by the Subject of Academic priority discipline of Jiangsu Higher Education Institutions (ZYX03KF027).
